# Current practices in peer review in German radiation oncology: a nationwide survey

**DOI:** 10.1007/s00066-025-02444-6

**Published:** 2025-07-24

**Authors:** Andrea Baehr, Maja Guberina, Philipp Ernst, Kilian Koch, Marion Juretko, Ursula Nestle, Maximilian Grohmann

**Affiliations:** 1 Department of Radiation Oncology, University Hospital Hamburg-Eppendorf, Hamburg, Germany; 2 Department of Radiotherapy, University Hospital Essen, West German Cancer Center, University Duisburg-Essen, Essen, Germany; 3 Department of Radiation Oncology, Kliniken Maria Hilf GmbH, Moenchengladbach, Germany; 4 Department of Radiation Oncology, MVZ Klinikum Straubing GmbH, Straubing, Germany; 5 Department of Radiation Oncology and Radiotherapy, University Medical Center, Johannes Gutenberg University, Mainz, Germany

**Keywords:** Quality assurance, Patient safety, Radiation planning, Radiation oncology, Education

## Abstract

**Background:**

Clinical peer review (PR) is a structured process in which medical professionals evaluate the quality of their colleagues’ work to ensure compliance with healthcare standards. In radiation oncology (RO), intra-institutional PR has become established as a key quality assurance (QA) measure to improve treatment safety and effectiveness. While various guidelines and recommendations exist internationally, no uniform PR framework for radiation treatment decision-making and planning has been defined in Germany.

**Objective:**

This study aims to provide an overview of current PR practices in German RO departments, assess the degree of implementation of recommended PR measures, and identify areas for improvement.

**Methods:**

A digital survey among RO specialists was conducted from January 7 to February 7, 2025. The survey included structured questions on PR implementation, participation of different professional groups, timing, documentation, and technological infrastructure. Free-text fields allowed for additional insights. The collected data were analyzed descriptively.

**Results:**

A total of 51 complete questionnaires—mainly from academic centers—were evaluated. Here, PR was widely implemented, particularly involving physicians and medical physicists, with 86% of departments performing case discussions and 82% PR of plans before the first radiation session. Most participants reported that PR effectively supports treatment planning and safety. However, PR for target delineation and image fusion was only implemented in 41% of cases. The inclusion of RTTs, nurses, and radiologists was rare. Documentation of PR processes, particularly attendance tracking and implementation of recommended changes, was inconsistent. Time constraints, personnel shortages, and high patient volume were the most frequently reported barriers to continuous PR.

**Conclusion:**

While PR seems to be an integral part of radiation therapy in Germany, its structure and implementation throughout centers need to be elucidated. While some aspects, such as pre-therapeutic PR, are well established in our cohort, gaps remain in the integration of multidisciplinary teams, structured documentation, and contouring reviews. Further discussions within the German RO community and the development of national recommendations could help to standardize PR processes and improve their efficiency and effectiveness.

## Introduction

Clinical peer review (PR) is defined as a process by which physicians evaluate the quality of work performed by their colleagues for the purpose of determining compliance with appropriate standards of health care [1]. The German Medical Association (*Bundesärztekammer*) defines PR as follows:

“*Ärztliches Peer Review ist definiert als kritische (Selbst‑)Reflexion des ärztlichen Handelns im Dialog mit Fachkollegen – unter Verwendung eines strukturierten Verfahrens mit dem Ziel einer kontinuierlichen Verbesserung der Qualität und Sicherheit der Patientenversorgung.*”

(English: “Medical peer review is defined as critical (self-)reflection of the physician’s actions in dialogue with specialist colleagues—using a structured procedure with the aim of continuously improving the quality and safety of patient care”) [2].

With regard to radiation oncology (RO), intra-institutional PR has been defined as a cornerstone of safe treatment in several statements and recommendations [3–6]. Works like the ASTRO 2013 statement emphasize its importance for all professional groups before and during radiation planning as well as during radiotherapy delivery [4]. Peer review reduces errors in planning and treatment concepts before they can affect patients and develop into real incidents [7–9]. Besides, the overall quality of planning and RO concepts improves through a consistent culture of PR, which standardizes concepts and enhances staff knowledge [10, 11]. Implementation of PR in the form of conferences (chart rounds) to review treatment plans and target volumes is widely adopted, though variations exist in frequency, timing, participants, and content [9, 12–14].

Despite the existence of guidelines in other countries addressing this topic [4, 15–17], no uniform approach to PR in German RO has been reported or implemented so far. The aim of this survey was to assess current approaches to PR in German RO. Furthermore, with a unified German definition of PR applicable to RO, our group seeks to provide a foundation for further research and recommendations.

## Methods

The German Society for Radiation Oncology (DEGRO) working group on patient safety derived recommendations concerning PR in RO leaned toward several international recommendations, including those from professional societies such as ASTRO and RCR, as well as toward safety and quality improvement frameworks in clinical settings [4–6, 16, 18]. A medical physicist and a senior physician in RO screened these publications for recommendations concerning PR for treatment intent and planning. Based on this, recommendations were developed in three 1–2‑h meetings of our working group with 5–8 participants each. These were included in an online questionnaire using LimeSurvey (LimeSurvey GmbH, Hamburg, Germany). All recommendations, quotes, and references are detailed in Table 1.

**Table 1:** Recommendations, references, and corresponding response rates (in %). The total number of completed questionnaires was 51. Percentages not summing to 100% indicate missing responses

Category	Recommendation	Quote	Implemented (%)	Partly implemented (%)	Not implemented (%)
Attendance	Various professional groups are present at meetings to discuss the case and treatment concept	Where it is practical and reasonable, peer review sessions generally should include broad members of the clinical team and should not be limited to physicians [4]	69	20	10
	*Physicians* are present during discussions of the case and treatment concept	Where it is practical and reasonable, peer review sessions generally should include broad members of the clinical team and should not be limited to physicians [4]	96	2	2
	*Medical physicists* are present during discussions of the case and treatment concept	Where it is practical and reasonable, peer review sessions generally should include broad members of the clinical team and should not be limited to physicians [4]	69	16	14
	*RTTs* are present during discussions of the case and treatment concept	Where it is practical and reasonable, peer review sessions generally should include broad members of the clinical team and should not be limited to physicians [4]	27	31	35
	*Nurses* are present during discussions of the case and treatment concept	Where it is practical and reasonable, peer review sessions generally should include broad members of the clinical team and should not be limited to physicians [4]	6	18	71
	At least two physicians are present during the peer review of treatment plans	QA meetings should include at least two clinical oncologists (or peer professional with appropriate competencies) with relevant site-specific expertise. In some departments this will necessitate the meeting being linked by videoconferencing [16]	57	31	10
	At least one physician who is not directly involved in the case is present during the peer review of treatment plans	Peer review meetings should therefore mandate the presence of at least one non-treating physician with expertise in the tumor site subspecialty, in order to provide objective feedback on treatment planning [18]	63	22	14
	*Resident physicians* are also present during the peer review of treatment plans	It is desirable for such a meeting to also be attended by oncologists in training, radiologists, physicists, and dosimetrists/radiographers [16]	59	12	25
	*RTTs* are also present during the peer review of treatment plans	It is desirable for such a meeting to also be attended by oncologists in training, radiologists, physicists, and dosimetrists/radiographers [18]	22	22	55
	*Radiologists* are also present during the peer review of treatment plans	It is desirable for such a meeting to also be attended by oncologists in training, radiologists, physicists, and dosimetrists/radiographers [18]	4	8	80
	Participation in peer review-based measures/meetings is mandatory	Participating in peer review efforts should be required and could be motivated by considering such participation during workers’ evaluations [4]	55	20	22
Content	All cases are presented in a peer review-based discussion with regard to clinical background and the proposed treatment concept	Details of each patient’s evaluation and intent for treatment are briefly presented to the other radiation oncologists and clinical team and are used as early peer review for the basic treatment decisions and plan [6]	63	24	10
	The presentation of the therapeutic concept includes the treatment goal (palliative vs. curative), radiation technique, dose concept, and details on IGRT and movement management	The general approach includes parameters such as a) treatment goal (e.g., curative, adjuvant, or palliative); b) treatment modality (e.g., brachytherapy vs. various external beam approaches); c) non-conformal or conformal, image-guidance approach (e.g., cone beam); and d) motion management (e.g., gating) [4]	73	20	4
	The fusion with imaging and the segmentation of the target volumes are presented in a peer review-based discussion	Peer review of segmentation/fusion (as well as the images chosen for segmentation) should ideally be done prior to treatment planning, as much of the subsequent work is dependent on the details of the segmentation and/or fusion [4]	41	35	18
	When presenting target volumes, the presenting college explains how the volumes were created. Special features/uncertainties are highlighted	The oncologist who defined the volume (principal clinician) should ideally be present to explain how the TVs have been defined, though these details should also be recorded on the planning note. Areas of uncertainty should be highlighted and discussed [16]	35	45	14
	Target volumes for curative therapies that a) have been individualized and thus deviate from the usual contours in the institution b) that follow a new protocol are peer reviewed	Prospective peer review […] included the following: all individualized volumes; any protocol-specified volume that does not conform to the department protocol; any protocol-specified volume defined within a new protocol where the volume is different to that used previously. Prospective review should continue until adequate audit shows that the new protocol is being followed appropriately [16]	57	24	14
	Target volumes for palliative therapies that are associated with a high degree of complexity, for example in the case of preload, are reviewed by means of peer review	Prospective peer review […] included the following: all individualized volumes; any protocol-specified volume that does not conform to the department protocol; any protocol-specified volume defined within a new protocol where the volume is different to that used previously. Prospective review should continue until adequate audit shows that the new protocol is being followed appropriately [16]	71	16	10
	Target volumes for which a change was decided in a previous meeting are presented again	Prospective peer review should be performed in situations where a clinically important difference in judgement between oncologists might occur.[…] The revised volumes should be subject to further peer review to ensure compliance with the recommended changes [16]	53	24	16
	For target volumes for palliative and curative therapies defined on the basis of the applicable standards (routine volumes), peer review-based discussions only take place for a limited number of all cases	For example, 10% of volumes could be randomly selected and audited at a peer review meeting. As random errors in a complex process are unpredictable, including some of these volumes in prospective peer review is recommended. Retrospective audit of volumes should be performed for a) protocol-specified volumes that are defined according to protocol and b) routine palliative radiotherapy treatments [16]	61	14	22
	For pre-irradiated patients, the approved dose in organs at risk and target volumes is decided in a peer review-based meeting	Retreatment settings are often particularly challenging (especially when normal tissue constraints in the target volume are compromised) and represent a clinical setting where peer review of this decision is often useful [4]	65	27	4
	The plan quality and the clinical feasibility of the plan are checked by means of peer review	Peer review for planning evaluates two types of decisions from the planning process: the physician-driven clinical tradeoffs (ideally addressed through physician peer review) and the more technical aspects of the plan’s ability to achieve the desired dose-volume results with reasonable complexity and deliverable fields (ideally addressed through dosimetrist/physicist peer review) [4]	73	18	4
Content	Changes in the therapeutic goal or changes in the anatomy during treatment are checked by peer review	Similarly, changes in the planning goals or image segmentation made during the course of therapy (i.e., adaptive therapy) may also benefit from peer review [4]	55	33	8
	The department has defined contouring standards for all entities against which disputed/complex cases can be compared	When critically appraising target volume definition within the peer review setting, it is recommended that centers determine the standard guideline or protocol against which each case is being compared. This also helps to ensure that centers maintain up-to-date knowledge of recent developments in contouring recommendations [18]	47	35	16
Organization and equipment	The spatial and technical conditions enable an effective and efficient peer review	Leadership needs to […] provide the necessary infrastructure for efficient and effective peer review (e.g., adequate space, image display capabilities, access to electronic records, support staff to help monitor and facilitate review, software tools) [4]	80	10	8
	During the plan presentation, the plan is shown for all to see (projector/screen)	Meaningful peer review often requires that the planning images, diagnostic images, and electronic medical records (EMRs) be readily accessible.[…] Multiple projectors with multiple screens may be needed so that groups can review planning images, diagnostic images, and the EMR concurrently [4]	80	10	8
	When presenting the case and treatment concept, diagnostic images, surgical reports, and histologies are available and can be shown on screens or projectors	Meaningful peer review often requires that the planning images, diagnostic images, and electronic medical records (EMRs) be readily accessible. Computers and workspaces should be outfitted to view multiple sources of data concurrently [4]	76	16	6
	The objectives of peer review-based meetings have been clearly and precisely defined	The specific goals and targets of peer review should be clearly specified, and the results of each peer review effort should ideally be tracked [4]	61	20	14
	Procedural instructions for peer review have been clearly and precisely defined	Practices should standardize policies and procedures for this review [5]	59	16	22
	There is a standard for the peer review of target volumes in the department	Each department should have an agreed process for peer review of target volumes [4]	48	25	22
Frequency and schedule	The peer review-based presentation of cases and planned treatment concepts takes place before the first radiation treatment	The first series of process steps are components of medical decision-making that come before the treatment planning process. […] Optimal peer review for these items is performed prior to treatment planning as changes in these items will influence the planning process [18]	86	6	4
	The peer review-based evaluation of treatment plans takes place before the first treatment session	Peer review should take place before the first fraction of radiotherapy is delivered [16]	82	8	4
	The peer review-based assessment of target volume contouring is carried out for complex cases before the plan is drawn up	We therefore recommend that peer review for simpler cases be carried out for TVD and plan review after physics/dosimetry input, and for more complex cases, peer review should focus on TVD alone to ensure agreement on treatment volumes before planning begins [18]	67	25	2
	When new treatment-relevant information becomes available, relevant peer review processes are repeated (e.g., when staging studies have been updated)	The timing of this peer review should be pretreatment and during the planning process, as much of the information needed to judge a decision becomes available only after the planning process has been initiated [4]	63	25	6
	Given the number of cases and their complexity, the department has defined a standard for the type of cases that are routinely presented in a peer review-based meeting	Individual radiotherapy centers should clearly categorize which cases should routinely undergo peer review, taking into account their caseload and expected case complexity, in order that sufficient time is allocated to case discussion [18]	51	12	31
	In order to enable a meaningful discussion of all cases, a maximum of 10 cases are discussed per hour	Radiotherapy departments should realistically expect no more than 10 cases per hour to be scheduled [16]	29	22	43
Documentation	The results of peer review meetings are documented	When peer review is performed it should be documented. Documentation also acts as a checklist to ensure all relevant metrics have been reviewed. The document should include a record of the participants and of any changes made to TVs, radiotherapy doses or concomitant treatments. The terms “major change” and “minor change” are suggested to help provide some quantification [16]	61	24	10
	Attendance at peer review meetings is documented	The document should include a record of the participants and of any changes made to TVs, radiotherapy doses, or concomitant treatments. The terms “major change” and “minor change” are suggested to help provide some quantification [16]	37	20	39
	A standardized form is used for the documentation of peer reviews	Radiotherapy departments should use a standardized peer review outcome record template which will facilitate audit of their processes in relation to QA, peer review of target volumes, review of amendments in target volumes after peer review, and review of radiotherapy treatment plans [16]	41	0	51
	Whether decisions from peer review-based meetings have been implemented is recorded and documented	The specific goals and targets of peer review should be clearly specified, and the results of each peer review effort ideally should be tracked [4]	41	14	39

A definition of PR was provided, encompassing all professional groups, with examples to aid understanding and survey completion:

“Peer review corresponds to a reflection of medical action by several specialist colleagues, often including multiple professional groups (example: discussion of planned radiation concepts in conferences; position control on the radiation machine; review of radiation plans).”

(German: “*Peer Review* *entspricht einer Reflexion des medizinischen Handelns durch mehrere Fachkolleg:innen, dabei gehören zu dieser Personengruppe oft mehrere Berufsgruppen (Beispiel: Besprechung geplanter Bestrahlungskonzepte in Fallbesprechungen; Lagekontrolle am Bestrahlungsgerät; Überprüfen von Bestrahlungsplänen).*”)

A digital survey was available from January 7 to February 7, 2025. The invitation link was sent via the mailing list of the DEGRO and shared on LinkedIn (LinkedIn Corporation, Sunnyvale, CA, USA). Participation was anonymous, and all participants consented to data evaluation and publication.

Participants were asked to rate the extent to which specific recommendations concerning PR were implemented in their departments on a three-point scale: implemented, partly implemented, and not implemented. To avoid incorrect information due to lack of knowledge, none of the items were mandatory. Free-text fields were provided for each item.

## Results

Fifty-one completed questionnaires were eligible for evaluation. Table 2 shows details of department types, the types of treatment cases, and the number of cases per year, as reported by the participants in this survey. Table 2 presents all results, along with the respective recommendations and citations.

**Table 2:** Characteristics of the participants’ institutions. The total number of completed questionnaires was 51

Question	Response option	Absolute number (*n*)	Percentage (%)
Department type	University hospital	18	35
	Hospital	4	8
	Private practice	13	25
	Combination	16	31
Types of cases	Only outpatient cases	4	8
	Outpatient and hospital cases	47	92
Number of patients treated annually	Up to 500	3	6
	500–1000	10	20
	1000–2000	18	35
	More than 2000	20	39

### Attendance in peer review

Participants were asked about attendance in PR for pretreatment planning (such as dose prescription, etc.) as well as in PR of radiation plans. Only one participant reported that no PR takes place in their department. Most participants reported that physicians, and to a lesser extent medical physicists, at least partly attend PR for treatment intent, treatment concept, and modality. In contrast, non-academic professionals such as RTTs and nurses participate in PR meetings significantly less often: RTTs are regularly involved in only one third of all departments, and 71% of departments reported that they do not include nurses in these activities. According to free-text responses, time constraints limit the participation of physicists and RTTs in regular concept meetings. Some departments reported that at least one representative from each professional group, such as a lead person, attends each meeting. A total of 55% of participants reported compulsory attendance in PR within their department. No significant differences were observed between the different types of departments for this category.

For quality assurance of treatment plans, two thirds of participants reported that either at least two physicians in total or at least one physician who was not previously involved in the case attend the review meetings. Residents regularly participate in plan review in 59% of cases. Several free-text comments provided additional insights. Some participants stated that all physicians—including residents—attend the reviews, whereas in some departments, only special cases are brought to larger PR conferences. Others reported a PR approach conducted solely by a physician–physicist pair. Regarding residents’ attendance, some participants reported that they do not have residents in their department, which applies particularly to private practices. Most participants reported that RTTs are not integrated into PR of treatment plans. Furthermore, radiologists are generally not included, whereas some radiation oncologists have undergone specialty training in radiology and thus serve as both radiology and RO specialists.

### Content of peer review

Patient cases are commonly presented in PR-based conferences in 63% of participants’ departments. Among them, 73% reported that case presentations contain details on therapeutic intent (palliative or curative), dose prescription, radiation technique, and motion management. Free-text responses showed that the choice of irradiation technique is often made later during the course of planning, for example by medical physicists, and the details regarding image-guided RT (IGRT) are rarely discussed in these meetings. Peer review of radiation plans regarding quality and feasibility is commonly implemented (73%). The approach to review of plans seems to differ: some institutions only use PR for complex planning, others evaluate the plan quality with two persons or in larger conferences.

The fusion of diagnostic imaging with the planning CT, along with the presentation of the corresponding delineated structures, is included in PR in only 41% of cases. A total of 57% of participants reported that volumes for curative treatments involving new protocols or deviations from established concepts are regularly reviewed, whereas most indicated that in routine cases, only a minority of volumes are presented for PR. In contrast, PR of palliative contouring is common in 71% of participants’ departments when cases are complex, such as in patients undergoing re-irradiation. In 35% of departments, volumes are presented along with explanations of specific challenges encountered during contouring. Only 47% of participants reported that their institution has contouring guidelines that allow for a standardized comparison of all reviewed volumes.

Free-text comments reveal a variety of approaches on target definition review: some participants reported that all volumes are reviewed by multiple physicians, whereas in other departments, PR is only implemented for complicated cases. Requested changes in volumes lead to a second review in 53% of participants’ institutions on a regular basis. Some participants indicated that standards exist for certain organs or that their local standard operating procedures (SOPs) reference previously published guidelines.

A total of 65% of participants review the recommended doses for re-irradiated patients in all cases, while 27% do so for a subset of cases, either before planning or during plan revision.

When new relevant information, such as new staging imaging, becomes available, 65% of participants reported that PR is repeated. However, free-text responses show that repeated PR is typically conducted only when the new findings lead to plan modification. Similarly, changes in anatomy or changes in the treatment intent during the course of therapy are regularly addressed in PR in only 55%. Multiple comments highlight a wide variety of implementation approaches: some participants report on repeated discussions during plan review, in other departments, the decision appears to be at the discretion of the responsible senior physician.

### Organization and equipment

Concerning equipment, 80% of participants rated their spatial conditions and equipment as sufficient for efficient PR, and radiation plans are commonly presented for all participants on screen (80%). Diagnostic imaging, other relevant diagnostics, or patient history are available in 78% of cases; however, free-text responses showed that these are not commonly presented. The scope and standards of PR conferences have been defined in 61% of departments. While some participants reported having established SOPs, others described the processes as being based on “common knowledge,” without a written definition.

### Frequency and schedule

Peer review for clinical concepts and planning review is typically conducted before the first radiation session in 86% and 82% of participants’ departments, respectively. For complex cases, contouring is reviewed before treatment planning begins on a regular basis in only 24% of departments, while an additional 26% conduct reviews on demand.

A local standard for selecting which cases are presented in PR-based conferences based on complexity and timing exists in 51% of the surveyed departments. However, multiple free-text responses indicated that some institutions discuss every case in PR. A limitation of case discussions to a maximum of 10 per hour is not widely implemented (29%), but appears more common in non-university hospitals or private practices (*p* = 0.063). Repeated case discussions when new relevant diagnostic findings become available are conducted in 63% of departments. No significant differences between department types were observed in this category.

### Documentation

A total of 61% of participants reported that PR results are routinely documented, with 41% using a standardized form. Some participants described electronic documentation or brief notes in patient records, while others reported that PR is documented without details on its content. Documentation of attendance is consistently implemented in only 37% of cases and varies widely between departments. In some institutions, attendance is compulsory for all physicians and therefore assumed for all cases, while in others, senior physicians sign all plans during review. Whether a recommended change has been implemented is systematically recorded in only 41% of participants’ departments.

## Discussion

Peer review is an efficient tool for preventing errors in RO, and several expert committees have issued statements on the subject [19, 20]. However, in Germany, no official recommendations exist for PR in treatment decision-making and radiation planning. Analogous to the procedure for other QA measures, such as the planning of certain techniques or machine-based QA, international recommendations can serve as a reference. This may result in a wide range of approaches [21]. Even though our evaluation showed that PR is widely used in German RO, the degree to which the aspects of PR are characterized varies, as was to be expected based on results from other countries [22, 23]. This variability also reflects findings from Fairchild et al., who reported wide differences in RT protocol compliance in multicenter trials [24]. Given that non-compliant RT was associated with poorer clinical outcomes in multiple studies, strengthening PR frameworks could contribute to improved standardization and potentially better patient outcomes. A large survey in the MENAT Region revealed that implementation of more sophisticated techniques correlated with PR activities [22]. The authors also found that PR was valued for education, adherence to guidelines, improving planning protocols, and reducing variation in practice institutions without PR. Interestingly, none of the recommendations were more consistently implemented in larger hospitals or practices, implicating that departmental organization rather than the size of the team might have an impact here.

The most pronounced implementation in our investigation was shown for the recommended time sequence of case discussion and plan approval and first radiotherapy, which took place consecutively in 86% and 82%, respectively. This is particularly noteworthy as in the US, plan discussions often take place during the first week of radiotherapy [14], despite multiple studies showing that the rate of comments and changes to radiotherapy plans depends significantly on the timing of PR during the course of treatment [14, 25, 26]. In this context, the consistent adherence to the recommended sequence is encouraging, whereas the full implementation of PR for radiotherapy plans only appears to be possible in 73% of cases and in only 63% for treatment concepts. Even if there appears to be room for improvement here, this could exceed expectations in an international context, as, for example, Brundage et al. reported prospective PR in only half of all Canadian RO centers for curative cases, with even lower rates in provincial centers [23, 27].

Several societies and authors have endorsed the importance of PR for treatment intent or planning by implementing respective quality and safety indicators, and Canada in the meantime even tracks the implementation rate of prospective PR for RO planning [17, 28, 29]. Reddemann et al. were able to show that it is possible to increase the rate of PR with a structured program and changed management approach [30]. Moreover, PR activities could also be enhanced by creating multi-institutional networks [31]. The role of leadership in this process has been highlighted in multiple recommendations [5, 6]. A key factor in successful PR implementation is the availability of adequate facilities, equipment, and procedural frameworks. Our data show that the equipment was considered as appropriate for PR. The review of plans and cases is ensured by presentations on screens and beamers. In contrast, the objectives and processes for PR have been defined in only 60% of departments. Similarly, documentation remains uncommon, particularly regarding the use of predefined forms or templates. Attendance records and whether recommended changes were then implemented are also rarely documented. Several studies have emphasized the importance of standardized procedures and clear objectives in ensuring high-quality processes in healthcare, such as the WHO’s High 5s project [30]. Furthermore, the clear documentation of quality assurance processes was recommended by various authors, including the AAPM [4, 5, 33], and was shown to have a significant impact on the quality of healthcare [34].

Contouring and registration seems not to be a common part of PR in German RO departments. This is particularly noteworthy since various authors have shown a high inter-observer variability for target volumes, with an impact on the quality of treatment [35, 36]. In contrast, regular PR of volumes can be expected to result in reduced variability over time, as shown by data from the USA, for example [31]. The joint PR of contours is also a decisive point for training, which should increase the quality of treatment at the center in the long term. In this respect, the participation of residents in PR activities is also recommended [11, 37], but our results show that this is not translated in common practice. A key barrier to educational benefits may be the low rate of structured presentations on contouring challenges during conferences (35%) and the limited availability of standardized contouring guidelines in many institutions.

Competences of RTTs are not commonly part of case or plan reviews, and nurses are only part of PR of treatment intent in a minority of departments. This certainly indicates a limitation in the comprehensive implementation of pretherapeutic PR, as Kotecha et al. demonstrated that multidisciplinary meetings, including all professional groups, led to notable changes in treatment intent. These changes primarily affected patient positioning but also influenced overall treatment concepts [38]. The competences of RTTs in radiation planning have been defined by the ESTRO Radiation Therapist Committee, including tasks such as volume delineation and dose–volume histogram (DVH) assessment, even at the undergraduate level [39], even though the authors recognize that the tasks for RTTs vary throughout member states. Unlike in other countries [40], RTT is not an academic profession in Germany. This structural difference, along with perceived professional boundaries between physicians, medical physicists, and RTTs, may hinder case discussions on an equal level. The role of nurses in PR is also not well defined, and some publications do not see a role for nurses here [41]. This is contrary to the role of the nurse as an advocate for patients, as it was stated by the Oncology Nursing Certification Corporation (ONCC) [42]. Nurses may have significant insight into the clinical status of patients, e.g., concerning their ability to tolerate positioning. This might be of advantage especially in palliative settings, as was shown in a similar way for interprofessional ward rounds in RO [43]. The inclusion of radiologists in PR of plans might have a big impact on planning [44] but was absolutely not common in our survey, and the free-text answers indicated that the reason for having radiologists in plan PR is because physicians have undergone double-specialist education.

Barriers to continuous PR were numerously reported in our free-text responses and seem to comprise lack of time, lack of personnel, and high patient numbers. This goes along with findings from other countries [22, 23]. It was found that only a minority of departments have a regular fixed concept regarding which cases are presented at all, in view of the short time available. Even though plan review is a powerful tool for intercepting errors [19], prospective studies have shown that only a fraction of the errors are detected [45]. In the referenced study, the effectiveness in recognizing errors was highest during the first 30 min of the meeting. In an editorial regarding the value of chart rounds, the authors postulated that a high number of cases leads to apathy among participants, which significantly reduces effectiveness [14].

If the size of a practice makes it more difficult to bring together multiple professional groups and medical colleagues for PR meetings, the option of remote participation should be considered. This approach has been successfully implemented by Riegel et al. [46]. For departments with a particularly high throughput, digital formats or special applications for structuring the meeting can at least increase efficiency and possibly also effectiveness [47]. However, it is important to recognize that the use of digital solutions may also influence the dynamics and outcomes of PR [48].

## Limitations

In the context of the methodology with an anonymous survey, it is not possible to exclude that some answers may derive from the same institution. Moreover, the quantity of 51 answers can only reflect a part of the reality in all nearly 300 German RO departments. We saw the majority of answers deriving from university hospitals, which represent a minority of departments in the real world. Besides, our results may bear bias, as primarily persons who are more interested in quality assurance measures in their institutions took part in the investigation. For this reason, our results do not allow for any generalization to clinics and practices of different sizes and could also present a distorted picture due to the predominance of rather progressive institutions among the participants.

Nevertheless, this study offers a first insight into the field of PR, which has not yet been systematically analyzed for German radiotherapy. It highlights key areas for further research and may serve as a foundation for future studies and potential recommendations to standardize and optimize PR processes in Germany.

## Conclusion

This study provides a first overview of PR practices related to treatment intent and planning in German RO. Our findings indicate that, at least in the presented cohort, PR is implemented, particularly involving physicians and medical physicists and that the recommended sequence of pretherapeutic PR is commonly followed. Additionally, staff generally report sufficient spatial conditions and equipment for PR.

However, PR for target delineation and image registration as well as the inclusion of RTTs, nurses, or radiologists remains uncommon. Furthermore, there is room for improvement in the structured organization and documentation of PR processes. Our working group strongly encourages further discussion within the German RO community, and future recommendations should support departments in optimizing the efficiency and effectiveness of PR activities.
